# An Ishmaelite

**Published:** 1888-05-26

**Authors:** Leslie Keith

**Affiliations:** Author of "The Chilcotes," "Uncle Bob's Niece," "East and West," etc.


### An Ishmaelite.

By Leslie Keith,

Author of "The Chilcotes," "Uncle Bob's Niece," "East and West," etc.

Chapter VIII.—In the World once More.

"Can any of us understand what it means," Arthur had said. Those of us who have escaped the deepest pitfalls and not paid the direst penalty for our lapses—can we understand what five years of banishment mean, and what liberty means at the end of that term?

Fred left his youth behind him in Dartmoor. He was but twenty-seven when he came out, but already he was a middle-aged man, grey and haggard; all the hopefulness, and brightness, and gaiety gone from him, never to return. We have mercifully substituted remedial punishment in our thoughts, for that sad old doctrine that decreed the unrepentant sinner to eternal misery in another life; but in this life punishment does not always purify. The prison left its taint on Fred; it hardened him in some points and it weakened him in others. The moral nervelessness that allowed him to fall now paralysed his hope of rising. Arthur met him ready for the fight, ready to stand by his side and do battle with him against all the world; and he found him unvaliant, acquiescent in his doom.

Arthur never described that meeting. The secret of it remained deep in his heart, but for days he could not look at his cousin without suffering an ache there. He took him down to a quiet little seaside place where nobody knew them, and where Fred might gather courage to face the world he had come back to.

"They mercifully let you grow your hair for a week or two before the bolts are drawn," he said, with that melancholy bitterness that always afterwards coloured his speech. "Perhaps the village folks won't discover me, Arthur, but I feel as if I had the livery on yet. Shall I ever get rid of the feeling? Boy, boy, why did you come to me? Don't you know that the taint will stick to me till I die? Better have forgotten me. I'm not fit company for gentlemen now."

"Don't, don't," groaned Arthur. "You'll break my heart. I've counted the months till this day; I've thought of nothing else."

Fred stretched out a hand across the table.

"So there's a good Samaritan left in the world, is there? Tell me about—about the people I used to know ——"

He drew back his hand and shaded his face while Arthur began falteringly and with many pauses to fill up the outline of those blank years in Fred's record. When he spoke of his Aunt Helen and gave her last whispered message of love, a tear crept through Fred's fingers and splashed on the table. Arthur's voice was husky, and he could scarcely go on. He passed from that sad topic as quickly as he might; but Fred's face was still hidden when he told of Nellie's marriage. He escaped from that, too, to touch with a reactionary lightness on Mr. Mack's offer, and to set it forth with all the humour he could command.

"He's a good old fellow, and he means very well," he said. "But I've a much finer scheme for us than that. I've no fancy for martyrdom in the shape of malodorous niggers, and fevers, and alligators, and mosquitos, whatever you may have. We've a world to choose from."

Fred looked up at last.

"You've told me of my mother, whom I killed, and of Nellie, whose love I killed too. You haven't said a word of my father; I know what that means. I have no father, and he has no son. He cares no more what becomes of me than he troubles himself about the mongrel that slinks at night to the shelter of his doorstep. It is left to old Mack, whom I wronged the most, to hold out his hand in sign of peace and pardon."

Arthur hung his head. He had no contradiction to give.

"Do you think I wasn't prepared for it?" said Fred, half bitterly, half fiercely. "Do you suppose a man has not time in prison to think? It is his thoughts that are his curse. I used to pray that I might lose my memory, but I never could forget that I was dead—dead to him, and to all of you. I don't complain. It's no more than I've deserved."

"Have I forsaken you?" said Arthur very sadly. "Are you going to send me away, Fred? You may send me, but I will not go. I will stick to you whether you want me or not."

Fred looked at him with a melancholy smile.

"Suppose I were to accept your sacrifice," he said. "Suppose I were to let you give up all your prospects in London; give up the girl you love, perhaps, if you have found one to love—your friends and companions, at any rate—and go with you across the seas, how long do you think my story would be in following us? We should find it on that other shore awaiting us. What sort of a welcome would it prepare for us? Do you think they love the scum out there because England ships it over to them? They love their honour too, and for such as I am they have nothing but contempt and scorn. I've spent a fifth part of my life behind prison walls, but I know better than you, my boy, in what esteem the world holds criminals."

"Do you think I care for its scorn and contempt, even if the world be what you call it, a monster of heartlessness and cruelty? and I don't believe it," cried Arthur. "Isn't there such a thing as forcing it to respect you? Is a man to be shut out for ever because of one stumble? Are we to set ourselves up to condemn where God himself does not condemn?"

"We haven't got to heaven yet, if there is a heaven, and while we are on earth it's by the world's precepts we must go. It has decreed that for those of us who fall there is no climbing up again."

"It may be by a hard way, but it's to be done."

"Not by me. What have I to gain that I should face the struggle? Haven't I lost all I prized already—the love of the two best women in the world, and my own self-respect? The day I slew that, Arthur, I buried my ambition with it. If I must live on—and I accept it as part of my punishment—I will live in some corner where I shall offend no one's susceptibilities and hurt nobody's morals. There are worse people perhaps than I in the world. I will go among them."

It was in vain that Arthur argued, entreated, insisted—put forth all the advantages of life in a new land, among new traditions. His fortune was at Fred's command; his friendship, his unwavering faith and belief were Fred's for ever. Fred listened, but his determination remained unshaken.

"My life is done," he said. "I finished my career when the doors of Millbank closed upon me; I will drag out what remains of my term in some corner where I shall not stink in anybody's nostrils."

Arthur was by turns abashed and indignant before this spectacle of morbid weakness, but he could not inspire it with a better courage. Fred had never had much manliness or moral stamina, and he was but obeying an instinct natural to him when he refused to fight, and preferred to hide his wounds in obscurity.

What thoughts and vain regrets and passionate longings visited him in those long, light, summer nights, when he could not sleep, and lay listening to the sobbing of the sea, starting sometimes from a fitful sleep at the fancied call of the warder, and remembering with scarce a feeling of relief that he was no longer a bondsman at the law's bidding! Once, when his restlessness became a torment, he rose and slipped into his cousin's room. The summer moonlight filled it, and illuminated Arthur's face. In sleep it looked very young and boyish, and singularly frank and open.

"If I could have fallen to such a depth of meanness as to accept his sacrifice," he said to himself, "I should deserve the worst the world could give me. Could I bear to see him day by day growing more disappointed, more hopeless, striving to hide the suffering he felt? You could never raise me to your side, Arthur, and, heaven helping me, I will not drag you down to mine."

Arthur stirred in his sleep and suddenly sat up.

"Did you call me, Fred?"

"I didn't mean to waken you, old fellow; a demon of restlessness took possession of me, and seeing your door open I strolled in."

"I could have sworn you called me; it was a dream, I suppose, but it was a very jolly one. We were out there, you and I," he pointed to a vague distance, "in the heart of a great lonely forest —— "

"I have had my vision, too," said Fred, stopping Arthur with a gesture of his hand. "Our forest will be London, if you like, Arthur—a man may be as lonely there as in the desert."

"You will come back to London? That is brave, Fred."

"Not your London, my boy; I have no place there now. But there are other Londons, where I may be as completely hidden as in your virgin forest, and yet where you may find me sometimes if you will, in the South or the East—— "

"The slums! Do you know the sort of people that live down there?"

"Do you know the sort of people to whom I belong? You will find many in the meanest courts of London who have the right to hold themselves high above me—the right that a clean score gives them, and yet I think down there they have a little more compassion for a brother's abasement than in the West."

"But what could you do?" said Arthur, greatly disturbed over this proposal. "There is no work for a gentleman to do down there."

"There are no gentlemen in Dartmoor," said Fred curtly. "We lose those fine distinctions. Look at my hands; you can see them even by this faint light; they have been taught to work. Find me, if you can, some obscure corner and some honest work to do. I have set you a harder task than you dream of, Arthur; you will discover that the gaol-bird is the true unemployed. All doors are shut to him."

"You've banished sleep, anyhow, and it is a shame to sleep on such a night as this. Come down to the sea, I'll dress in five minutes. Perhaps, with the salt wind in your face, you'll listen to reason."

But neither at night nor in the morning could Arthur prevail, and he finally gave in and set out with a heavy heart on his mission. "And what will you do meantime?"

"I'll stop here," said Fred with a bitter smile, "if they will suffer me. I am not likely to run away, and I haven't given the law a right to grab me again yet."

It was speeches like this that wounded Arthur, and sent him despondingly on his search.

He came back in a couple of days, looking as dull and gloomy as when he set out.

"I've found you something," he said savagely, "about as mean and miserable as you could wish."

"So soon?" said Fred. "You have been in luck, Arthur. Did you make me out a victim to a mistake, or what magic did you use? I'll spare you the recital of all the rebuffs, I can imagine them beforehand; but tell me of this wonder among men who is willing to hold out a helping hand to a released criminal. You told him, of course?"

"I told him," said Arthur, hanging his head and speaking huskily; I thought it best to start clear. He is a very decent fellow—a Scotchman; I knew him long ago, and haven't lost sight of him."

"A Scotchman! then the miracle is greater still; come, tell us all about it.

"It is a miserable place."

"The most miserable would be too good for me," said Fred sadly. "Speak up, Arthur; I will take it thankfully whatever it is."

*(To be continued.)*

